# Mutation Status and Immunoglobulin Gene Rearrangements in Patients from Northwest and Central Region of Spain with Chronic Lymphocytic Leukemia

**DOI:** 10.1155/2014/257517

**Published:** 2014-03-30

**Authors:** I. González-Gascón y Marín, J. A. Hernández, A. Martín, M. Alcoceba, M. E. Sarasquete, A. Rodríguez-Vicente, C. Heras, N. de las Heras, R. Fisac, A. García de Coca, I. de la Fuente, M. Hernández-Sánchez, I. Recio, J. Galende, G. Martín-Núñez, J. M. Alonso, J. M. Hernández-Rivas, M. González

**Affiliations:** ^1^Servicio de Hematología, Departamento de Medicina, Hospital Universitario Infanta Leonor, Calle Gran Vía del Este 80, 28031 Madrid, Spain; ^2^Universidad Complutense de Madrid, Avenida Séneca 2, 28040 Madrid, Spain; ^3^Servicio de Hematología, IBSAL-Hospital Universitario de Salamanca, Paseo de San Vicente 58-182, 37007 Salamanca, Spain; ^4^Centro de Investigación del Cáncer-IBMCC, Universidad de Salamanca (USAL-CSIC), Calle Zacarías González 2, 37007 Salamanca, Spain; ^5^Servicio de Hematología, Hospital Virgen Blanca, Calle Altos de Nava, s/n, 24071 León, Spain; ^6^Servicio de Hematología, Hospital General de Segovia, Calle Miguel Servet, s/n, 4002 Segovia, Spain; ^7^Servicio de Hematología, Hospital Clínico Universitario de Valladolid, Avenida Ramón y Cajal 3, 47005 Valladolid, Spain; ^8^Servicio de Hematología, Hospital del Río Hortega, Calle Dulzaina 2, 47012 Valladolid, Spain; ^9^Servicio de Hematología, Hospital Nuestra Señora de Sonsoles, Plaza de Santiago 1, 05002 Ávila, Spain; ^10^Servicio de Hematología, Hospital El Bierzo, Calle Médicos sin Fronteras 7, 24411 Ponferrada, León, Spain; ^11^Servicio de Hematología, Hospital Virgen del Puerto, Paraje Valcorchero, 10600 Plasencia, Cáceres, Spain; ^12^Servicio de Hematología, Hospital Río Carrión, Avenida Donantes de Sangre, s/n, 34005 Palencia, Spain

## Abstract

The aim of this study was to investigate the frequency and mutation status of the immunoglobulin heavy variable chain (IGHV) in a cohort of 224 patients from northwest and central region of Spain diagnosed with chronic lymphocytic leukemia (CLL), and to correlate it with cytogenetic abnormalities, overall survival (OS) and time to first treatment (TTFT). 125 patients had mutated IGHV, while 99 had unmutated IGHV. The most frequently used IGHV family was IGHV3, followed by IGHV1 and IGHV4. The regions IGHV3-30, IGHV1-69, IGHV3-23, and IGHV4-34 were the most commonly used. Only 3.1% of the patients belonged to the subfamily IGHV3-21 and we failed to demonstrate a worse clinical outcome in this subgroup. The IGHV4 family appeared more frequently with mutated pattern, similar to IGHV3-23 and IGHV3-74. By contrast, IGHV1-69 was expressed at a higher frequency in unmutated CLL patients. All the cases from IGHV3-11 and almost all from IGHV5-51 subfamily belonged to the group of unmutated CLL.

## 1. Introduction

Chronic lymphocytic leukemia (CLL) is a clinically and biologically heterogeneous disease, with survival times ranging from months to decades [1, 2]. Rai and Binet staging systems were designed to provide prognostic information, and are widely used in clinical practice [3, 4]. However, some patients with early stages rapidly progress.

Over the past decade, several biological markers have become important prognostic factors and may guide treatment decisions. These include immunoglobulin heavy chain variable region (IGHV) mutation status, expression of specific proteins on CLL cells such as CD38 and intracellular zeta-associated protein-70 (ZAP-70), and some cytogenetic abnormalities [5–9].

Clonal genomic aberrations can be identified in approximately 80% of CLL patients by fluorescence* in situ* hybridization (FISH). Usually, patients with deletion of chromosome 13q14 as a single alteration have a better outcome, although a high number of losses in 13q14 or large deletions including* RB1* gene could be associated with a worse outcome [10]. By contrast, patients with deletion of chromosome 11q22 or chromosome 17p13 show a shorter OS, while cases with trisomy 12 have an intermediate prognosis [7, 11].

The IGHV gene mutation status is one of the most reliable prognostic markers. It defines two different subsets of CLL based on the cutoff value of 98% identity with the closest germ line IGHV genes: mutated CLL (M-CLL) and unmutated CLL (U-CLL). Somatic mutations of IGHV occur in approximately half of the cases and usually present with nonprogressive disease, in contrast to patients with U-CLL who have a more aggressive disease with a shorter progression-free survival, time to first treatment (TTFT), and overall survival (OS) [11, 12]. Furthermore, irrespective of mutation status, some heavy chain variable regions have been associated with specific clinical features and varying occurrences from country to country. For example, IGHV1-69 gene has been observed to be one of the most frequently rearranged genes in Western patients and is almost always associated with the subset of U-CLL [13]. Other subgroups reported to be frequently used in Western patients are IGHV3-23, IGHV4-34, and IGHV3-07. Moreover, an overrepresentation of the IGHV3-21 gene has been reported in northern European countries compared to the Mediterranean region, and it has been associated with a worse prognosis despite the mutation status [13, 14].

In this study, we investigated the frequency and mutation status of IGHV in a cohort of patients from northwest and central region of Spain. We also analyzed the relationship between IGHV mutation status and some other CLL prognostic markers such as the expression of CD38, cytogenetic abnormalities detected by FISH, OS, and TTFT.

## 2. Patients, Materials, and Methods

### 2.1. Patients

A total of 224 unselected patients diagnosed with typical CLL from nine different institutions located in northwest and central region of Spain were studied for IGHV gene usage and mutation status. The diagnosis was based on clinical symptoms, immunophenotypic analysis, blood cell count, and cell morphology, according to the World Health Organization classification of tumors [15] and National Cancer Institute guidelines [16]. The study was approved by the local ethics committee and all individuals provided their informed consent. The median age of the patients was 63 years (range, 29–90 years). A hundred and fifty-two patients were male (68%) and seventy-two (32%) were female (male/female ratio = 2.1). At the time of diagnosis, most cases were classified as stage A (77%) according to Binet classification [4].

### 2.2. Sample Processing and DNA Extraction

Mononuclear cells were isolated using Ficoll density gradient centrifugation from peripheral blood samples containing more than 10% of CLL cells determined by flow cytometry. Genomic DNA was extracted and purified, washed, and lysed according to the manufacturer's instructions using the DNAzol kit (Molecular Research Center, Cincinnati, OH, USA).

### 2.3. PCR Amplification of IgH Rearrangements

IGHV gene rearrangements were amplified by reverse transcription-PCR according to ERIC recommendations [17], using a mixture of 5′ primers specific for framework region (FR) 1 consensus family (IGHV1-IGHV6), together with 3′ primers complementary to the germ line JH regions. All samples were tested for the amplification of IGH rearrangements according to the BIOMED-2 Concerted Action protocols in whose standardization our group has participated actively [18]. In this strategy, complete V-D-J rearrangement amplification was performed by multiplex PCR with a set of family-specific primers of the framework region (FR1) and one IGHJ consensus primer. When amplification was unsuccessful, a second PCR was performed with primers from the IGHV leader region. In all cases amplification consisted of an initial denaturation step of 10 minutes at 95°C followed by 35 cycles of 94°C, 60°C, and 72°C for 30 seconds each, with a final extension step of 30 minutes at 72°C.

### 2.4. Sequencing of PCR Products

Clonality was assessed by size discrimination of PCR products using automatic ABI 3130 Genetic Analyzer (Applied Biosystems, Foster City, CA, USA) in conjunction with GeneMapper 4.0 software (Applied Biosystems). To purify amplified products, they were run on 8% polyacrylamide gel, heteroduplexed in cases with polyclonal background, and visualized by ethidium bromide staining. Purified PCR products were then eluted from polyacrylamide gel and sequencing was performed from both directions, at least from 2 PCR reactions, using the BigDye Terminator v1.1 Cycle Sequencing Reaction Kit (Applied Biosystems) on an automated DNA Sequencer analyzer (ABI 3130, Applied Biosystems).

### 2.5. Analysis of IG Gene Sequences

The obtained sequences were aligned to V-Base (http://www2.mrc-lmb.cam.ac.uk/vbase/list2.php) data base. IGHV sequences with <98% homology with respect to the germ line counterpart were considered as mutated, while those with an identity greater than or equal to 98% were considered as unmutated. The cutoff value of 96% was also contemplated in order to analyze differences with possible prognostic interest.

### 2.6. FISH Analysis

Interphase FISH was performed on peripheral blood samples obtained at diagnosis using commercially available probes for the following regions: 13q14; 12q13; 11q22/ATM; 17pTP53; and 14q32/IGH (Vysis/Abbott Co., Downers Grove, IL, USA). Methods for FISH analysis are described elsewhere [19]. The sensitivity limit for the detection of 14q32 translocation, trisomy 12, and deletions was >3%, >3%, and >8% interphase cells with split signal, three signals, and one signal, respectively. Dual color FISH using differently labeled control probes was performed, and signal screening was carried out on at least 200 cells with well-delineated signals. Hybridization was repeated in those slides with less than 80% cells showing tow control-probe signals.

### 2.7. CD38 Expression

CD38 expression was analyzed by 4-colour fluorescence—activated cell sorting (FACS) analysis. For data analysis, the Infinicyt software (Cytognos SL, Salamanca, Spain) was used [20].

### 2.8. Statistical Analysis

Statistical analysis was performed using the SPSS 17.0 software package (SPSS, Chicago, IL, USA). The Fischer's exact test and the Chi-squared test were used to determine the relationship between categorical variables. Quantitative variables were compared by using the Student's* t-*test and the Mann-Whitney* U* test. OS was calculated from the time of diagnosis to death or last follow-up visit. TTFT was calculated as the interval between diagnosis and the start of first line treatment. OS and TTFT were estimated by the Kaplan-Meier method and assessed by the log-rank test. Statistical significance was defined as *P* < 0.05.

## 3. Results

### 3.1. IGHV Mutation Status

We analyzed mutation status of 224 patients with CLL. Based on a cutoff value of 98% nucleotide sequence homology of isolated IGHV gene with that of the germ line counterpart, a total of 125 (55.8%) cases were classified as M-CLL and 99 (44.2%) patients as U-CLL. When we considered 96% as the cutoff value, 102 (45.6%) cases displayed somatic mutations, whereas 124 (54.4%) were unmutated.

### 3.2. IGHV Gene Family Usage and Mutation Status

IGHV gene usage was identified in 216 patients. The most frequently used IGHV family was IGHV3: 107 (49.5%), followed by IGHV1: 55 (25.5%), IGHV4: 41 (19.0%), IGHV5: 8 (3.7%), IGHV2: 3 (1.4%), and IGHV7: 2 (0.9%), with no expression of IGHV6. Mutation status (≥98% homology) and its relationship with sex and IGHV rearrangements are summarized in Table 1.

IGHV3 and IGHV4 regions were found predominantly in the M-CLL cases (72/107, *P* = 0.004, and 30/41, *P* = 0.022, resp.) in contrast to IGHV1 genes, which were found predominantly in the U-CLL cases (37/55, *P* = 0.0001). Only 3.2% of patients used IGHV5 regions, and 7 out of 8 cases displayed unmutated status (*P* = 0.023).

Among IGHV1 family, IGHV1-69 was also preferentially expressed in U-CLL (18 versus 3 cases *P* < 0.0001).

**Figure 1:**
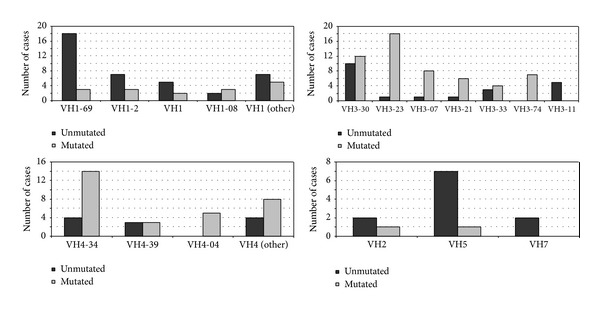
IGHV gene segments usage profile of patients with M-CLL and patients with U-CLL (number of cases).

Regarding IGHV3 family, IGHV3-30 was the most common region used (20.6%), followed by IGHV3-23 (19%), with a low representation of IGHV3-7 (4.0%) and IGHV3-21 (3.2%). IGHV3-21 and IGHV3-7 expressed a mutated status in all but one case. Both IGHV3-23 (*P* < 0.0001) and IGHV3-74 (*P* = 0.018) presented more frequently the mutated pattern. By contrast, the five patients with IGHV3-11 belonged to the subgroup of U-CLL (*P* = 0.016). Interestingly, within IGHV5 family, IGHV5-51 genes appeared to be mostly unmutated (*P* = 0.016).  Figure 1 shows IGHV gene segment usage profile of patients with M-CLL and patients with U-CLL.

### 3.3. Association of Mutation Status and Rearranged IGHV Genes with Disease Outcome

Patients with U-CLL seemed to have a more aggressive disease. Despite establishing homology in 98 or 96%, U-CLL patients presented with a higher initial white blood cell count (WBC) (median 31.5 × 10^9^/L versus 21.2 × 10^9^/L; *P* = 0.038) and higher levels of LDH (*P* = 0.003), belonged to advanced Binet stages (*P* = 0.001), progressed more frequently (*P* < 0.0001), and had a shorter leukocyte duplication time (*P* = 0.001). Diffuse pattern of bone marrow infiltration and secondary tumors (*P* = 0.047) were also more often observed in this subgroup of patients.

**Figure 2:**
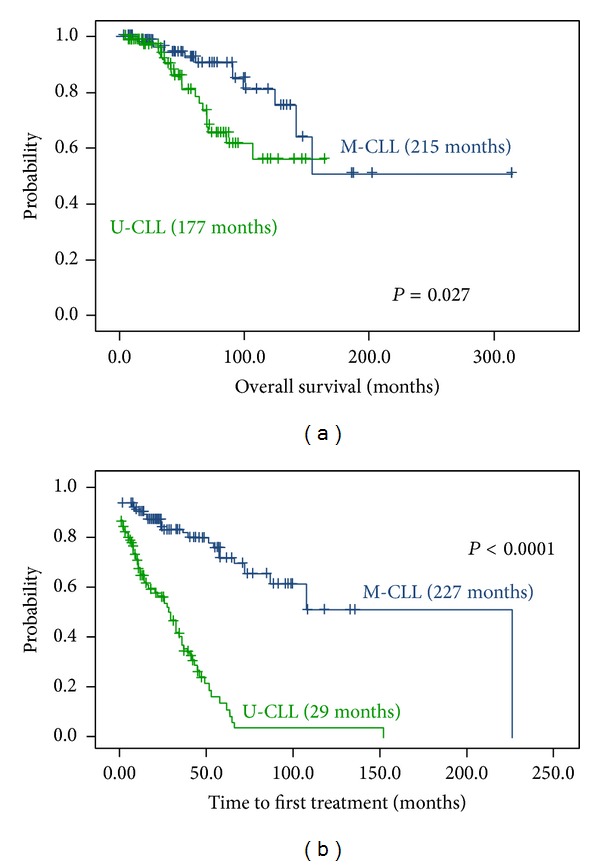
Kaplan-Meier survival curve comparing OS and TTFT between patients with M-CLL (125 cases) and U-CLL (99 cases). (a) Median OS for U-CLL: 117 months; median OS for M-CLL 215 months. The difference is significant at the *P* = 0.027 level (log-rank test). (b) Median TTFT for U-CLL: 29 months; median TTFT for M-CLL: 227 months. The difference is significant at the *P* = 0.0001 level (log-rank test).

The estimated median OS of the group with 98% or greater IGHV homology was 215 months, while the estimated median OS time of the subgroup with IGHV homology less than 98% was 117 months (*P* = 0.027) (Figure 2(a)). Comparison of TTFT between M-CLL and U-CLL revealed a significantly higher TTFT in M-CLL versus U-CLL (227 and 29 months, resp., *P* < 0.0001) (Figure 2(b)).

Regarding subfamily usage, it is noteworthy that patients with IGHV1-69 had a higher probability to be treated (*P* = 0.002) and developed more frequently secondary neoplasms (*P* = 0.005). By contrast, patients with IGHV3-07 were associated with a lower WBC count (*P* = 0.001), male sex (*P* = 0.03), and nonprogressive disease (*P* = 0.043). IGHV3-30 usage was associated with normal levels of LDH (*P* = 0.005). Patients expressing IGHV3-11 subfamily had poor or intermediate prognosis cytogenetic alterations in all cases but one including 17p deletion in two patients, l1q deletion in one patient, and trisomy 12 in two other patients.

We failed to correlate the use of IGHV3-21 region with any feature, probably due to the low representation of this subfamily.

Finally, it is remarkable that all patients who expressed IGHV5-51 were males (*P* = 0.048), presented more frequently with positive direct antiglobulin test (*P* = 0.028), and six out of seven showed cytogenetic aberrations including 11q deletion (2 cases), trisomy 12 (2 cases), and 14q32 translocation (1 case).

OS was significantly worse in patients expressing IGHV1-69 (*P* = 0.021) and IGHV3-11 (*P* = 0.025). Significantly differences in prognosis could not be found in other IGHV subgroups, including IGHV5-51 (*P* = 0.057). Regarding patients using IGHV4 family, they had a significantly higher TTFT (*P* = 0.001), with no cases of Richter syndrome in patients with IGHV4-39. Only 29/103 patients expressing IGHV3 received treatment (*P* = 0.016); albeit patients from subfamily IGHV3-21 showed a tendency to be treated earlier. On the other hand, 6/7 cases from family IGHV5 required treatment during followup (*P* = 0.001).

### 3.4. Genomic Aberrations, CD38, and Mutation Status

Patients with 13q deletion belonged significantly more frequently to the mutated group (*P* = 0.005). By contrast, high-risk aberrations such as 11q and 17p deletions were significantly associated with the unmutated group (*P* = 0.003 and *P* = 0.007, resp.). Patients with 12q trisomy were also associated with the unmutated group (*P* = 0.003). No relationship between patients without genomic aberrations and mutation status was observed (Table 2).

Data for the expression of CD38 was available for 121 patients. Fifty-six patients were CD38 positive and 65 were negative at a cutoff level for CD38 of 30%. A significant different expression of CD38 between patients with M-CLL (20) and those with U-CLL (36) was observed (*P* = 0.0001) (Table 2).

## 4. Discussion

In patients with CLL, the presence of a correlation between unmutated IGHV status and poor survival has been well established in several studies [7–9]. This has led to extensive investigations on the mutational status and IGHV gene usage in different populations of patients diagnosed with CLL over the last few years. Results from these studies suggest that the presence of ethnic variations and geographic background may affect IGHV gene usage, in CLL [21–25].

The aim of our study was to investigate the frequency and mutation status of IGHV in a cohort of 224 patients from northwest and central region of Spain and to correlate it with cytogenetic abnormalities, CD38 expression, OS, and TTFT.

We found 56% cases of M-CLL and 44% of U-CLL when we established homology cutoff value on 98%. These findings are consistent with previous publications in Western countries [9, 12, 24]. Our data also confirmed that patients with unmutated IGHV genes had a distinctly more malignant disease and much shorter OS and TTFT than those with somatic mutations.

IGHV gene usage family distribution was also comparable to those observed in Western countries [26], which showed a higher representation of the IGHV3 family followed by IGHV1 and IGHV4 (48.4%, 24.9%, and 18.6%, resp.).

With regard to the IGHV3 family, IGHV3-30 was the most frequently used segment followed by IGHV3-23. By contrast, IGHV3-7 was present in a relatively low frequency (4%) and was seen preferentially in M-CLL (8/9). Similar observations have been reported previously in Mediterranean countries and Serbian patients [12, 24, 27].

IGHV3-21 has been reported to be overrepresented in Scandinavian patients with a lower frequency in Southern European countries and has been associated with poor prognosis independent of mutational status [27, 28]. We only found 3.2% of the cases with this gene usage and 6 out of 7 belonged to the subgroup of M-CLL. Anyway, probably due to the underrepresentation of this gene, we could not see a worse clinical outcome in these patients, although a trend to a shorter TTFT was observed (*P* = 0.07). Studies from Asia [21, 22], South America [23], and Europe [12, 26] showed a low representation of IGHV3-11 subfamily, with a similar proportion of M-CLL and U-CLL in this subgroup. However, the Ukrainian group showed that its presence was associated with an increased risk of developing autoimmune hemolytic anemia [25]. Interestingly, in our cohort, IGHV3-11 gene was significantly overused in the unmutated group and was associated with a lower OS (*P* = 0.025), but we failed to find a relationship with the risk of developing autoimmune hemolytic anemia. Further study of larger series needs to be performed to validate this data.

IGHV1-69 is the most frequently used gene in IGHV1 gene family of Western populations, exhibiting in most of the cases a germ line profile [29] and, consequently, a more aggressive disease. The higher usage of IGHV1-69 has been reported in Ukrainian [25] and Mediterranean [12] CLL patients. In accordance with these observations, IGHV1-69 was also the most frequently used segment of IGHV1 family in our studied population and the second most commonly detected in the global series (21 cases, 9.4%). We could also confirm the association of IGHV1-69 with U-CLL cases, as 18 of our 21 cases had the unmutated profile (*P* < 0.0001), with a significant worse OS (*P* = 0.021).

IGHV4 family has been reported to be overused in the mutated subgroup of CLL [26]. In keeping with these findings, we confirmed the significantly preferential use of IGHV4 family in M-CLL (*P* = 0.02) and the consequently better outcome of this subgroup with a longer TTFT (*P* = 0.001). IGHV4-34 had previously been reported to be among the most frequently used genes in CLL rearrangements [12]. In our cohort, IGHV4-34 was used in 8.3% of the patients, similar to previously described in Mediterranean patients [30].

Regarding the remaining genes, we found a low representation of IGHV5, IGHV2, and IGHV7 gene families and lack of expression of IGHV6, as also described in other studies, regardless of their ethnic origin [23]. However, it is remarkable that, within the IGHV5 family, IGHV5-51 was used in 8 patients (3.2%); all of them were male and presented with a shorter TTFT (*P* = 0.01). We could not confirm that these patients had a worse prognosis, probably due to its low representation. Further series including more patients with this subfamily should be studied in the future to clarify these findings. The Serbian cohort [24] and the Mediterranean cohort [12] are the ones with more representation of this subfamily and showed only 2% of this usage. Contrary to our findings, they did not find differences in prognosis and mutation status in this subgroup, probably due to the low representation of this usage. Nevertheless, further studies of larger series need to be performed.

Del(13q), del(11q), +12, del(17p), and IGH rearrangement have been proved to be the most common and prognostically relevant chromosomal changes in CLL. As previously reported [9], we confirmed that del(13q) was the most prevalent genetic abnormality and was found more often in patients with M-CLL, confirming its more favorable clinical outcome. Our data also showed an association between high-risk aberrations del(11q), +12, and del(17p) and U-CLL, which is consistent with their poor clinical outcome [31].

CD38 has also been reported as an independent predictor of prognosis for CLL, and its high expression has been correlated to unfavorable outcomes. It is controversial whether high levels of CD38 expression may be used as a surrogate marker of mutation status [22]. However, in our study we found an association of CD38 and U-CLL.

## 5. Conclusions

Our data confirm that IGHV mutation status is one of the most significant molecular predictors for CLL prognosis. The identification of IGHV gene segments can provide additional information regarding clinical course of CLL. The use of IGHV3-21 had a low incidence in our series. IGHV3-11 identified a group of patients with poor prognosis. IGHV5-51 revealed a group of patients with a trend to a worse outcome. In addition, OS and TTFT were significantly related to IGHV mutation status. Further studies of larger series, preferably in the context of prospective clinical trials, need to be performed to validate our data.

## Figures and Tables

**Table 1:** Mutation status (≥98% homology) and its relationship with IGHV rearrangements and sex.

IGHV family	Mutated cases	Unmutated cases	Mutated and unmutated	%	*P*	Male/female
IGHV1	2	5	7	3.2	ns	4/3
IGHV1-02	3	7	10	4.6	ns	5/5
IGHV1-03	2	2	4	1.9	ns	0/4
IGHV1-08	3	2	5	2.3	ns	3/2
IGHV1-18	2	2	4	1.9	ns	4/0
IGHV1-46	3	1	4	1.9	ns	3/1
IGHV1-69	3	18	21	9.7	<0.0001	15/6
Total IGHV1	**18**	**37**	**55**	**25.5**	**<0.0001**	**34/21**
IGHV2-01	1	0	1	0.5	ns	0/1
IGHV2-05	0	1	1	0.5	ns	0/1
IGHV2-70	0	1	1	0.5	ns	1/0
Total IGHV2	**1**	**2**	**3**	**1.4**	**ns**	**1/2**
IGHV3	3	2	5	2.3	ns	3/2
IGHV3-07	8	1	9	4.0	ns	9/0
IGHV3-09	2	2	4	1.9	ns	2/2
IGHV3-11	0	5	5	2.3	0.016	4/1
IGHV3-13	1	2	3	1.4	ns	3/0
IGHV3-15	4	2	6	2.8	ns	2/4
IGHV3-20	1	1	2	0.9	ns	1/1
IGHV3-21	6	1	7	3.2	ns	5/2
IGHV3-23	18	1	19	8.8	<0.0001	12/7
IGHV3-30	12	10	22	10.0	ns	17/5
IGHV3-33	4	3	7	3.2	ns	5/2
IGHV3-48	1	2	3	1.4	ns	3/0
IGHV3-49	2	2	4	1.9	ns	1/3
IGHV3-53	1	0	1	0.5	ns	1/0
IGHV3-64	0	1	1	0.5	ns	1/0
IGHV3-72	2	0	2	0.9	ns	2/0
IGHV3-74	7	0	7	3.2	0.018	5/2
Total IGHV3	**72**	**35**	**107**	**49.5**	**0.004**	**85/22, ** **P** = 0.088
IGHV4-b	2	2	4	1.9	ns	1/3
IGHV4-04	5	0	5	2.3	0.051	3/2
IGHV4-30	1	1	2	0.9	ns	1/1
IGHV4-31	0	1	1	0.5	ns	1/0
IGHV4-34	14	4	18	8.3	ns	11/7
IGHV4-39	3	3	6	2.8	ns	3/3
IGHV4-59	2	0	2	0.9	ns	1/1
IGHV4-61	3	0	3	1.4	ns	2/1
Total IGHV4	**30**	**11**	**41**	**19**	**0.022**	**23/18**
IGHV5-05	0	1	1	0.5	ns	1/0
IGHV5-51	1	6	7	3.2	0.046	7/0
Total IGHV5	**1**	**7**	**8**	**3.7**	**0.023**	**8/0, ** **P** = 0.05
IGHV7-04	0	2	2	0.9	ns	0/2
Total IGHV7	**0**	**2**	**2**	**0.9**	**ns**	**0/2**

**Table 2:** Relationship between genomic aberrations, CD38, and mutation status.

FISH	Mutation status			
		M-CLL	U-CLL	*P*
11q deletion	Present	4	17	0.0001
	Absent	121	82	

13q deletion	Present	61	20	0.0001
	Absent	64	79	

17p deletion	Present	0	10	0.0001
	Absent	126	88	

12q trisomy	Present	10	22	0.0003
	Absent	115	77	

CD38*	Present	20	36	0.0001
	Absent	46	19	

*Performed in 121 patients.
